# Observational Management for Patients with Biochemical Recurrence Following Radical Prostatectomy, in the Absence of Detectable Disease on Restaging PSMA PET/CT Imaging

**DOI:** 10.3390/diagnostics16010032

**Published:** 2025-12-22

**Authors:** Katelijne C. C. de Bie, Jan-Jaap J. Mellema, Dennie Meijer, Frederik R. Teunissen, Pim J. van Leeuwen, Daniela E. Oprea-Lager, Maarten L. Donswijk, Roderick C. N. van den Bergh, André N. Vis

**Affiliations:** 1 Department of Urology, Amsterdam University Medical Center, VU University, 1081 HV Amsterdam, The Netherlandsa.vis@amsterdamumc.nl (A.N.V.); 2 Prostate Cancer Network The Netherlands, The Netherlands; 3 Cancer Center Amsterdam, Amsterdam University Medical Center, 1081 HV Amsterdam, The Netherlands; 4 Department of Oncology, Antoni van Leeuwenhoek Hospital—The Netherlands Cancer Institute, 1066 CX Amsterdam, The Netherlands; j.mellema@nki.nl; 5 Department of Radiation Oncology, Radboud University Medial Center, 6525 GA Nijmegen, The Netherlands; 6 Department of Urology, Antoni van Leeuwenhoek Hospital—The Netherlands Cancer Institute, 1066 CX Amsterdam, The Netherlands; 7 Department of Medical Imaging, Radboud University Medical Center, 6525 GA Nijmegen, The Netherlands; 8 Department of Nuclear Medicine, Antoni van Leeuwenhoek Hospital—The Netherlands Cancer Institute, 1066 CX Amsterdam, The Netherlands; 9 Department of Urology, Erasmus Medical Center, 3015 GD Rotterdam, The Netherlands

**Keywords:** prostate cancer, biochemical recurrence, negative prostate-specific membrane antigen PET/CT findings, observational management

## Abstract

**Background/Objectives:** In men with biochemical recurrence (BCR) after a radical prostatectomy (RP), salvage radiotherapy (SRT) is commonly recommended when imaging shows no metastases. The optimal management for patients with negative prostate-specific membrane antigen (PSMA) PET/CT findings at BCR remains uncertain. This study evaluated outcomes of patients with BCR and negative PSMA PET/CT to identify who may be safely observed and who may benefit from early SRT. **Methods:** This retrospective multicentre cohort study included 89 patients with BCR and negative PSMA PET/CT findings after a RP (2015–2022) who were managed with observation. The exclusion criteria were PSA levels ≥ 0.8 ng/mL at baseline, prior SRT, or prior or ongoing hormonal therapy. Minimum follow-up was 3 years. Biochemical progression (PSA rise > 0.2 ng/mL above baseline or initiation of additional treatment) and radiological progression (local or metastatic disease on follow-up PSMA PET/CT) were assessed. Patients were stratified by EAU BCR-risk classification. Multivariable Cox regression included age, biochemical persistence (BCP) after a RP, pathological tumour stage (pT), pathological ISUP grade group (pISUP), node status (pN), margin status (R), and PSA doubling time (PSAdt). **Results:** The median age was 66 years (IQR 60–69) and the median PSA measurement at BCR was 0.2 ng/mL (IQR 0.2–0.3). A total of 27/89 (30%) patients were EAU BCR low-risk and 62/89 (70%) were high-risk. At three years, biochemical progression occurred in 14/27 (52%) low-risk vs. 51/62 (83%) high-risk patients, with time to progression being 21 vs. 12 months (*p* = 0.01). A pISUP grade group ≥ 4 (HR 2.04 [95%-CI 1.11–3.74]; *p* = 0.022) and a PSAdt < 20 months (HR 5.72 [95%-CI 2.41–13,56]; *p* < 0.01) independently predicted biochemical progression. Radiological progression occurred in 43/68 (66%) rescanned patients, with 32/43 (74%) showing disease outside the prostatic fossa. **Conclusions:** Nearly half of patients with BCR and negative PSMA PET/CT findings who were classified as EAU BCR low-risk remained progression-free at three years. These results support a risk-adapted approach, indicating that SRT may be deferred in selected low-risk patients.

## 1. Introduction

Prostate cancer (PCa) is the second most-diagnosed malignancy in men around the world, with its incidence continuing to rise every year [1]. The majority of cases are detected at an early, localised stage, with a favourable prognosis and low disease-specific mortality. Among curative treatment options, radical prostatectomy (RP) offers excellent long-term outcomes. However, up to 30% of patients experience biochemical recurrence (BCR), defined as two consecutive prostate-specific antigen (PSA) measurements ≥0.2 ng/mL within 15 years of surgery [2].

In men with BCR, the European Association of Urology (EAU) guidelines recommend salvage radiotherapy (SRT) to the prostatic fossa, provided that there is no evidence of distant metastases [3]. SRT has been shown to improve long-term outcomes, particularly when administered early [4,5,6]. However, SRT is not without its limitations. While potentially curative, it may constitute overtreatment in patients with indolent disease or undetected metastatic spread and it is associated with side effects: approximately 20% of patients experience significant late genitourinary toxicity (e.g., urinary incontinence or strictures) and up to 15% experience gastrointestinal toxicity (e.g., intermittent bleeding or diarrhoea) [7]. Furthermore, the EAU guidelines suggest that selected patients with favourable characteristics, such as a pathological International Society of Urological Pathology (pISUP) grade group < 4 and a PSA doubling time (PSAdt) > 12 months, may be suitable for observation rather than immediate intervention [8,9].

Importantly, all of the above studies demonstrating the benefits of SRT were conducted prior to the prostate-specific membrane antigen (PSMA) positron emission tomography/computed tomography (PET/CT) era. The introduction of PSMA PET/CT has transformed the management of PCa recurrence by detecting disease outside the prostatic fossa in up to 43% of men with rising PSA levels after a RP [10,11]. Its high sensitivity, even at PSA levels below 0.5 ng/mL, has had a significant impact on clinical decision-making and early treatment outcomes [10,12,13,14]. PSMA PET/CT is increasingly used to guide the administration of SRT and to help identify patients in whom SRT may be avoided or deferred [15,16,17,18,19,20]. However, it remains unclear whether PSMA PET findings translate into improved long-term oncological outcomes and whether treatment decisions should be based solely on PET imaging results.

Management of patients with BCR but negative PSMA PET/CT findings (i.e., those in whom no locoregional or metastatic recurrence is visualised) remains challenging, as the source of PSA production is unclear and the optimal treatment approach is uncertain. Some studies have reported improved outcomes with SRT in patients with negative PSMA scans, whereas others have shown that a substantial proportion of patients experience no further PSA progression after BCR diagnosis, suggesting that empirically offering SRT to the prostatic fossa carries a risk of overtreatment. No studies to date have identified which patients with negative PSMA PET/CT findings would benefit from early SRT and who could be safely monitored. Therefore, our aim was to determine which category of patients with BCR following a RP who have negative PSMA PET/CT findings would benefit from early SRT and who could be safely managed with observation.

## 2. Methods

### 2.1. Study Design and Data Collection

This retrospective multicentre cohort study included consecutive patients who underwent PSMA PET/CT imaging due to biochemical recurrence following a radical prostatectomy, had negative PSMA PET/CT findings, and were subsequently managed with observation. Patients were identified from three institutions in the Netherlands: Amsterdam University Medical Centres (AUMC), the Netherlands Cancer Institute (NCI), and Sint Antonius Hospital (SAZ), Nieuwegein, between 2015 and 2022.

Eligible patients had histologically confirmed PCa that was treated with a RP, with or without (extended) pelvic lymph node dissection ((e)PLND). Patients experienced BCR, defined as two consecutive PSA measurements of ≥0.2 ng/mL, or a single measurement of ≥0.5 ng/mL, following curative treatment. All included patients had negative PSMA PET/CT scans at the time of BCR restaging (e.g., no locoregional or metastatic recurrence visualised). Consequently, no immediate salvage treatment was initiated and patients were offered observational management. The exclusion criteria were as follows: any prior treatment for BCR; previous adjuvant or salvage radiotherapy to the prostatic fossa or whole pelvis; or prior or ongoing hormonal therapy. Additional exclusion criteria included a PSA level of ≥0.8 ng/mL at the time of restaging PSMA PET/CT scanning, a history of non-PSMA-expressing tumours, and evidence of metastatic disease detected by staging PSMA PET/CT scan or conventional imaging prior to a RP. Minimum follow-up was three years, with at least two PSA measurements taken after initiation of observational protocol. This study was approved by the institutional review board in each participating centre (VUmc2019.274, IRBd19182, R&D/Z23.104).

The data that was collected included patient age, initial PSA level, clinical tumour stage (cT), biopsy ISUP grade group (bISUP), and any relevant diagnostic imaging findings available at diagnosis. Pathological data included pathological tumour stage (pT), pISUP grade group, lymph node status (pN), and surgical margin status (R). Postoperative PSA kinetics were assessed, including biochemical persistence (BCP), defined as any detectable PSA after a radical prostatectomy, as well as all other PSA values and their corresponding measurement dates. At the time of BCR, restaging PSMA PET/CT findings were documented, along with the treatment decisions made during multidisciplinary team meetings. Follow-up data included repeat PSMA PET/CT imaging reports in cases of suspected disease progression and for the following of management plans, including the date and type of initiated treatment.

### 2.2. Objectives

The primary objective of this study was to identify the clinical and pathological risk factors for the biochemical progression of PSA in men with BCR and negative PSMA PET/CT findings who were managed with observational care. Biochemical progression was defined as a PSA rise >0.2 ng/mL above the PSA level at the start of observation, or the initiation of new treatment during follow-up. Radiological progression was defined as the detection of local recurrence or metastatic disease on follow-up PSMA PET/CT imaging. The secondary objective was to assess predictors of metastatic disease detected during follow-up.

### 2.3. PSMA PET/CT Imaging and Interpretation

PSMA PET/CT imaging was performed at the NCI, AUMC and SAZ using EARL-accredited PET/CT systems [21]. The indication for PSMA PET/CT was BCR. Radiotracers included [^68^Ga]Ga-PSMA-11, [^18^F]DCFPyL, and [^18^F]JK-PSMA-7, with acquisition protocols varying by centre. At AUMC, [^18^F]DCFPyL scans were acquired at 120 min post-injection using a Philips Ingenuity PET/CT scanner. At NCI, [^18^F]DCFPyL scans were acquired at 60 min post-injection and [^68^Ga]Ga-PSMA-11 scans at 45 min post-injection using Philips Gemini TF-II (Philips, Best, Netherlands) or Vereos Digital systems (Philips, Best, The Netherlands). At SAZ, [^68^Ga]Ga-PSMA-11 scans were acquired at 60 min post-injection using either Siemens Biograph (Siemens Healthineers, Forchheim, Germany) or Phillips Gemini scanners. [^18^F]-labelled tracers were synthesised at an on-site cyclotron (AUMC). [^68^Ga]Ga-PSMA-11 was produced using a GMP-compliant automated synthesis module at NCI, or via a semi-automated module using a ^68^Ge/^68^Ga generator (Eckert & Ziegler (Eckert & Ziegler, Berlin, Germany) and ITG (ITM Isotope Technologies Munich, Garching/Munich, Germany)) at SAZ. Scans covered an area of mid-thigh to skull base and were combined with low-dose CT (120–140 kV, 40–80 mAs) or diagnostic CT (130 kV, 110 mAs), with or without intravenous contrast. Image acquisition and reconstruction followed EARL standards, including corrections for scatter, decay, and random events [21,22].

Scans were reviewed by experienced nuclear medicine physicians and from 2021 scans were reported according to E-PSMA guidelines, assessing intraprostatic lesions or local recurrence (Tr+), as well as the number and location of metastases [22]. Lesions were considered ‘positive’ for metastases if they had an EANM PSMA visual score of 2–3 and/or CT findings were suggestive of PCa metastases [22].

### 2.4. Follow-Up

There was no predefined protocol for the observational management of patients with no visible disease present on PSMA PET/CT imaging. The decision to defer immediate treatment was made on a case-by-case basis, often through a shared decision-making process involving the clinician and the patient. In some cases, observational management was chosen due to significant comorbidities that made active treatment undesirable. In other cases, it was based on favourable disease characteristics, such as slowly rising PSA levels or BCR detected long after a radical prostatectomy, or on patient preference, particularly when concerns about treatment-related side effects were a factor. In more recent years, some patients have been managed expectantly in accordance with the EAU criteria for low-risk BCR (PSA doubling time (PSAdt) > 12 months and pISUP grade group 1–3) [8]. In this study, observational management was defined as not initiating immediate salvage radiotherapy or systemic therapy, with patients instead undergoing routine clinical follow-up and regular PSA monitoring. Follow-up PSMA PET/CT imaging was performed if PSA levels increased or if a rapid PSA doubling time (PSAdt) was observed, as determined by the treating urologist in accordance with multidisciplinary team consensus. If follow-up imaging revealed local recurrence or metastatic disease, patients were subsequently treated in accordance with EAU guidelines [3].

### 2.5. Statistical Analysis

The baseline characteristics are summarised descriptively. The continuous variables were reported as medians with interquartile ranges (IQRs) and the categorical variables were reported as frequencies and percentages. The group differences were assessed using the Mann–Whitney U test. The variables with a skewness greater than 0.5 were log-transformed before regression analysis. A binned graph analysis identified a PSAdt cutoff of 20 months for distinguishing a low risk of biochemical progression. Multivariable Cox regression was used to identify the predictors of biochemical and radiological progression-free survival, incorporating established prognostic factors (age, pT-stage, pISUP grade group, pN status, R status, and BCP). The postoperative PSAdt variants were compared in univariable analyses, with the highest-performing measure incorporated into the multivariable model. The clinical tumour characteristics and initial PSA measurements were excluded due to multicollinearity, as indicated by the variance inflation factor (VIF) values > 5. The follow-up time was defined as the interval from BCR to biochemical or radiological progression, initiation of new treatment, or the last recorded PSA measurement in the absence of progression. Patients were categorised into two predefined subgroups according to the EAU BCR-risk classification (high-risk and low-risk). Kaplan–Meier survival curves were generated and differences between subgroups were assessed using the log-rank test. The predictive performance of the PSAdt was evaluated using time-dependent receiver operating characteristic (ROC) analysis, with the optimal cutoff determined by the Youden index. Statistical analyses were conducted using SPSS^®^ version 28.0.1.1 (IBM Corp., Armonk, NY, USA) and RStudio^®^ version 4.3.2. A two-sided *p*-value < 0.05 was considered statistically significant.

## 3. Results

### 3.1. Baseline Characteristics

A total of 89 patients with BCR after a RP and no evidence of disease on restaging PSMA PET/CT imaging were included. All patients opted for observational management following shared decision-making. The baseline characteristics are summarised in Table 1. At study entry, the median age was 66 years (IQR 60–69) and the median initial PSA level at the time of RP was 9.4 ng/mL (IQR 7.3–14). A RP was performed for low-, intermediate, and high-risk PCa in 15/89 (17%), 34/89 (38%), and 40/89 (45%) patients, respectively. ePLND was performed in 55/89 (62%) patients, yielding pN1-status in 11/89 (12%) and pN0-status in 44/89 (50%) patients. Biochemical persistence was observed in 18/89 (20%) of patients.

At the time of BCR, median PSA level was 0.2 ng/mL (IQR 0.2–0.3). The median time to BCR was 23 months (IQR 11–58), and the median PSA doubling time (PSAdt) was 10 months (IQR 5.0–18). Based on the EAU BCR-risk criteria, 27/89 patients (30%) were classified as low-risk and 62/89 (70%) as high-risk. Median follow-up time after BCR was 63 months (IQR 43–53), as shown in Table 2.

### 3.2. Biochemical Progression

After BCR, 75/89 patients (83%) experienced further biochemical progression with a median time to progression of 13 months (IQR 9.1–23) (Table 3). Complete three-year follow-up data were available, with biochemical progression rates of 38% at one year, 65% at two years, and 73% at three years (Table 4). At four and five years, progression was seen in 61/75 (81%) and 42/50 (84%) patients, respectively.

Stratifying the cohort into EAU BCR-risk groups, progression was observed in 16/27 (59%) of low-risk patients and 58/62 (94%) of high-risk patients. Time to progression was longer in the low-risk group (a median of 21 months [IQR 13–35]) compared to the high-risk group (a median of 12 months [IQR 7.3–20]; *p* = 0.01), Table 3. Progression rates at one to five years were higher in the high-risk group (55%, 77%, 83%, 92%, and 94%) than in the low-risk group (11%, 37%, 52%, 58%, and 57%), Table 4.

### 3.3. Predictors of Biochemical Progression

**Figure 1:**
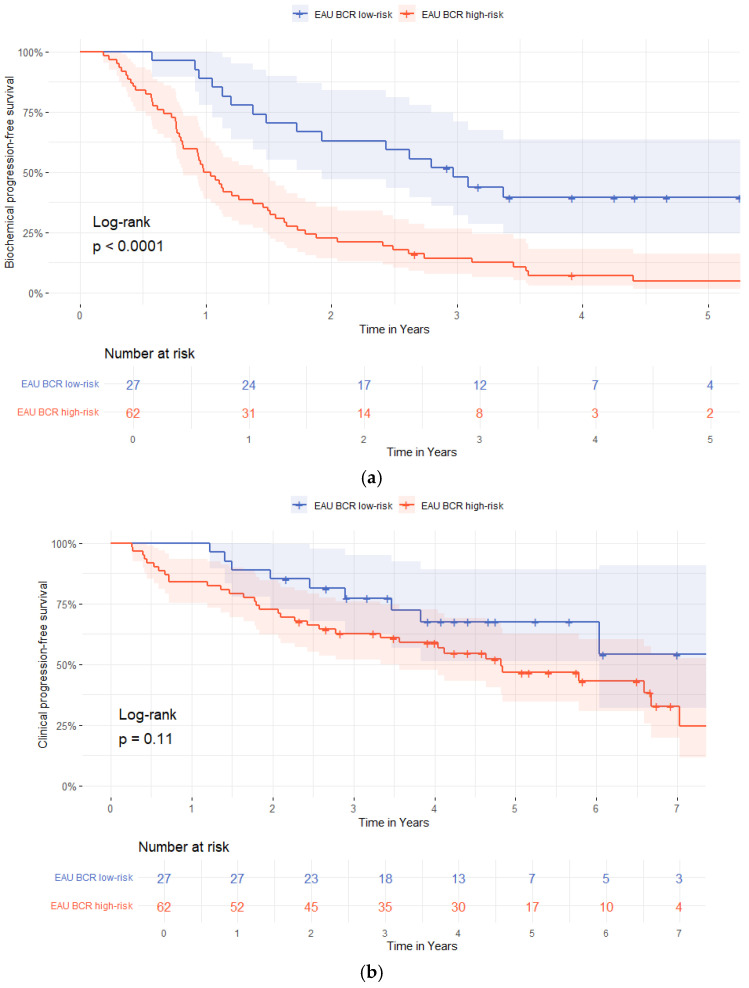

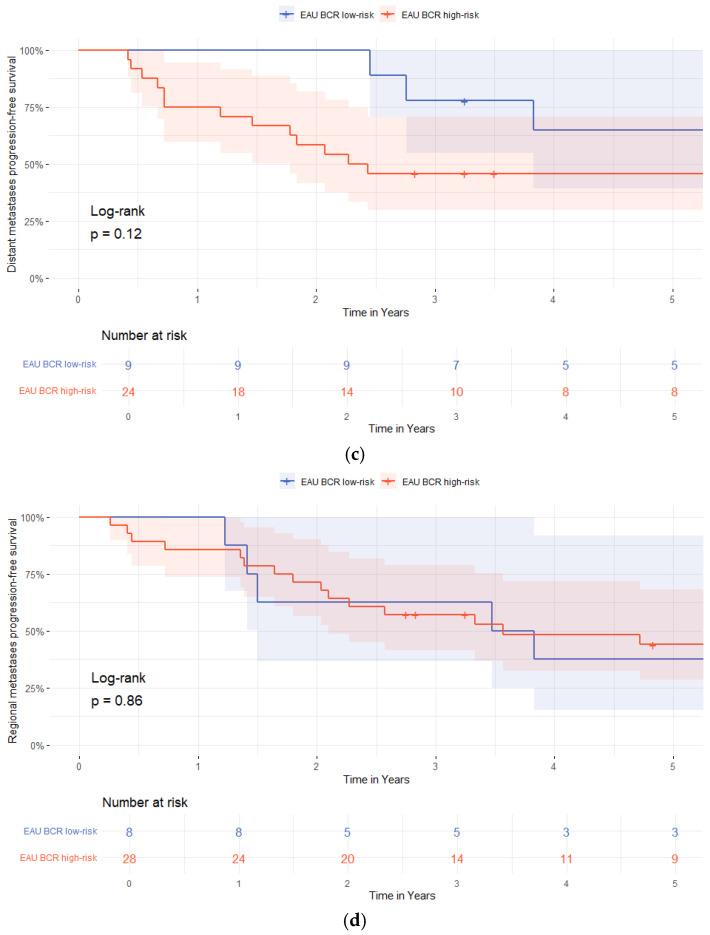
Kaplan–Meier survival curves stratified by European Association of Urology (EAU) biochemical recurrence (BCR)-risk group (low-risk vs. high-risk). (**a**) Biochemical progression-free survival; (**b**) radiological progression-free survival; (**c**) distant metastasis-free survival; (**d**) regional lymph node metastases free-survival.

In multivariable Cox regression analysis, adjusting for age, pT3-stage, pISUP grade group ≥ 4, pN-status, R1-status, BCP, and PSAdt to BCR ≤ 20 months, the independent predictors of biochemical progression after BCR were a pISUP grade group ≥ 4 and PSAdt to BCR ≤ 20 months (HR 2.04 [95%-CI 1.11–3.74]; *p* = 0.022 and HR 5.72 [95%-CI 2.41–13.56]; *p* < 0.01) (Table 5). Patients in the EAU BCR low-risk group had longer bPFS compared to those in the EAU BCR high-risk group (*p* = 0.04, log-rank), Figure 1.

### 3.4. The Role of Prostate-Specific Antigen Doubling Time After a Radical Prostatectomy

**Figure 2:**
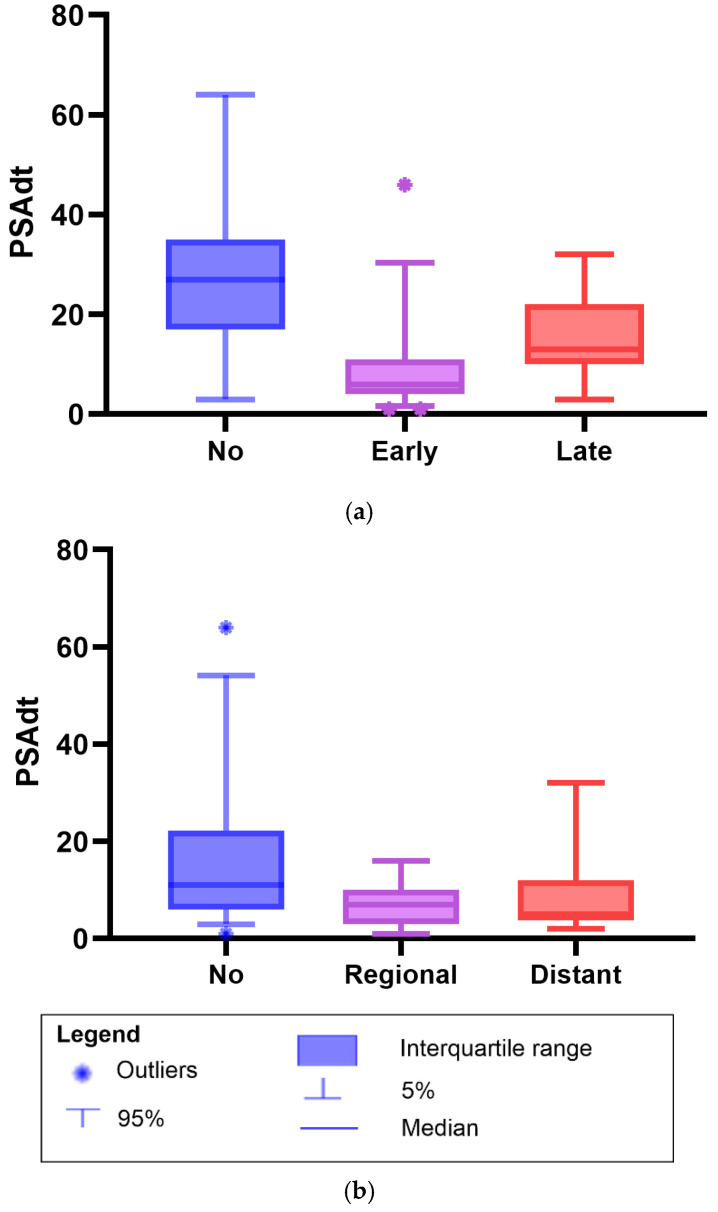
Boxplots of prostate-specific antigen doubling time (PSAdt) after radical prostatectomy in patients with biochemical recurrence with negative prostate-specific membrane antigen (PSMA) positron emission tomography/computed tomography findings managed with observation. (**a**) PSAdt in patients with early biochemical progression (1–2 years), late progression (3–5 years), or no progression. Median PSAdt: early progression, 6 months (IQR 4–11); late progression, 13 months (10–22); and no progression, 27 months (17–35). Mann–Whitney U test: early vs. no progression, *p* < 0.01; late vs. no progression, *p* = 0.03; and early vs. late progression, *p* = 0.01. (**b**) PSAdt by metastatic status on follow-up PSMA PET/CT imaging: no metastases, regional, or distant. Median PSAdt: no metastases, 11 months (IQR 6–22); regional, 7.0 months (3.0–10); and distant, 5.0 months (3.8–12). Mann–Whitney U test: no vs. regional metastases, *p* = 0.02; no vs. distant metastases, *p* = 0.01; and regional vs. distant metastases *p* = 0.88.

The PSAdt following a RP differed between patients with early biochemical progression (within 1–2 years) and those with late progression. The median PSAdt was 6 months (IQR 4–11) in the early progression group versus 13 months (IQR 10–22) in the late progression group (*p* = 0.01). In contrast, patients without further biochemical progression during follow-up had a longer median PSAdt of 27 months (IQR 17–35; *p*< 0.01) (Figure 2). Time-dependent ROC analysis demonstrated good predictive ability for late biochemical progression (AUC = 0.754, *p* = 0.01). The optimal PSAdt cutoff for predicting late biochemical progression was 9.0 months, yielding a sensitivity of 80% and specificity of 65% (Youden’s Index = 0.447).

### 3.5. Radiological Progression

PSMA PET/CT restaging was performed in 68/89 (74%) patients during follow-up, while 21/89 (26%) did not undergo subsequent imaging because of a non-rising PSA value, or a minimal PSA level rise deemed clinically insignificant by the treating urologist. Of the patients who underwent PSMA PET/CT restaging, 43/68 (66%) showed positive PSMA findings. Of these, 11/43 (26%) patients had local recurrence and 32/43 (74%) had disease outside of the prostatic fossa, including 12/43 (28%) patients with pelvic lymph node metastases and 20/43 (46%) with distant metastases. The median time to radiological progression was 25 months (IQR 14–46) and the median time to development of distant metastases was 22 months (IQR 8.6–33).

Radiological progression occurred in 9/27 (33%) patients in the EAU BCR low-risk group and in 34/62 (55%) patients in the high-risk group (Table 3). Time to radiological progression was not different between the groups (a median of 29 months [IQR 17–44] vs. 23 months (IQR 8.5–49]; *p* = 0.49). Distant metastases were detected in 4/27 (25%) low-risk and 16/62 (31%) high-risk patients, with longer time to detection in the low-risk patients compared to the high-risk patients (a median of 40 months [IQR 3–73] vs. 18 months [IQR 8.0–27]; *p* = 0.01), Table 3.

### 3.6. Predictors of Radiological Progression

In a multivariable Cox regression analysis adjusting for age, pT3-stage, pISUP grade group ≥ 4, pN-status, R1-status, BCP, and PSAdt to BCR ≤ 20 months, independent predictors of radiological progression after BCR were pT3 (HR 2.61 [95%-CI 1.23–5.51]; *p* = 0.0012), a pISUP grade group ≥ 4 (HR 4.45 [95%-CI 2.06–9.65]; *p* < 0.01), pN1 and pNx (HR 3.24 [95%-CI 1.16–9.11]; *p* = 0.025 and HR 2.26 [95%-CI 1.11–4.61]; *p* = 0.024), R1 (HR 2.39 [95%-CI 1.04–5.50]; *p* = 0.039), BCP (HR 2.80 [95%-CI 1.31–6.01]; *p* = 0.01, and a PSAdt to BCR ≤20 months (HR 3.44 [95%-CI 1.01–11.74]; *p* = 0.048), Table 6.

In addition, the median PSAdt was shorter in patients with metastases compared to those without; Figure 2. Patients without metastases had a median PSAdt of 11 months (IQR 6.0–22), compared to 7.0 months (IQR 3.0–10) in those with regional metastases (*p* = 0.02), and 5.0 months (IQR 3.8–12) in patients with distant metastases (*p* = 0.01). No difference was observed in the median PSAdt between patients who developed regional or distant metastases (*p* = 0.88).

Time-dependent ROC analysis showed poor discriminatory ability of PSAdt for predicting radiological progression (AUC = 0.27, 95%-CI 0.09–0.46, *p* = 0.04). Additionally, stratification by EAU BCR-risk group showed no association with metastasis-free survival for either regional (*p* = 0.86) or distant metastases (*p* = 0.12), Figure 1.

### 3.7. Treatment Changes

During follow-up, 59/89 (66%) patients received treatment for biochemical or radiological progression, with a median time to treatment change of 17 months (IQR 9.0–33). This differed by EAU BCR-risk group: a median of 31 months (IQR 19–45) in the low-risk group vs. a median of 15 months (IQR 9.0–30) in the high-risk group (*p* = 0.01). SRT was initiated in 27/89 (30%) patients and 14/89 patients (16%) received either pelvic- or metastatic-directed radiation therapy. Hormonal therapy (HT) was initiated in 18/89 (20%) patients after a median of 34 months (IQR 24–55), including 3/27 (11%) in the EAU BCR low-risk group and 15/62 (24%) in the high-risk group. Castration-resistant prostate cancer (CRPC) occurred in only the high-risk group (6/62, 10%), with a median time to CRPC of 49 months (IQR 33–67). Among patients who received additional treatment, the median end-of-follow-up PSA value was 0.1 ng/mL (IQR 0.0–0.7; range 0.0–1396.0). In patients who did not receive treatment, the median PSA value was 0.5 ng/mL (IQR 0.4–2.25; range 0.0–13.9), with 22/30 (73%) having PSA levels < 0.8 ng/mL.

## 4. Discussion

In men experiencing biochemical recurrence (BCR) following a radical prostatectomy (RP), the European Association of Urology (EAU) guidelines recommend salvage radiotherapy (SRT) to the prostatic fossa, provided there is no evidence of distant metastases. For patients with low-risk BCR (defined as a pathological International Society of Urological Pathology grade group (pISUP) < 4 and a prostate-specific antigen doubling time (PSAdt) >12 months), observational management is currently recommended. Notably, approximately 50% of patients scanned for BCR (at a PSA level < 0.5 ng/mL) demonstrate no detectable lesions on prostate-specific membrane antigen (PSMA) PET/CT scans [23]. Previous studies suggest that patients with BCR and negative PSMA PET/CT findings may be suitable for observation [17,24,25]. However, it remains unclear whether PSMA negativity indicates biologically indolent or early-stage disease, or alternatively, the presence of micro metastatic disease below the threshold of detection. This study aimed to identify which patients with BCR after RP who also have negative PSMA PET/CT findings may be safely observed and who may benefit from early SRT.

In our cohort of 89 patients with negative PSMA scans at BCR, 30% were classified as low-risk and 70% as high-risk according to the EAU BCR-risk classification. The median follow-up was 63 months (IQR 43–53) and 84% of patients experienced further biochemical progression. At a three-year follow-up, the biochemical progression rate was 52% in the EAU BCR low-risk group compared to 83% in the EAU BCR high-risk group. In multivariable analyses, independent predictors of biochemical progression were a pISUP grade group ≥4 (HR 2.04 [95%-CI 1.11–3.74]; *p* = 0.022) and a PSAdt to BCR ≤ 20 months (HR 5.72 [95%-CI 2.41–13.56]; *p* < 0.01). Radiological progression occurred in 48% of patients at a median of 25 months (IQR 14–46). The strongest independent predictors were a pISUP grade group ≥4 (HR 4.45 [95%-CI 2.06–9.65]; *p* < 0.01) and a PSAdt to BCR ≤20 months (HR 3.44 [95%-CI 1.01–11.74]; *p* = 0.048).

Our findings are consistent with those of Emmett et al., who prospectively studied BCR patients whose management was based on PSMA PET/CT findings [17]. In their cohort, 56/260 (22%) patients had negative PSMA scans (i.e., no prostatic fossa recurrence or metastases), of whom 29 received observational management. At three years, 19/29 (66%) patients experienced biochemical progression (defined as a PSA level rise >0.2 ng/mL). In our cohort, the three-year progression rate was similar, at 73%, but with clear differences between the EAU BCR-risk groups: 52% in the low-risk group versus 83% in the high-risk group. This suggests that combining PSMA PET/CT negativity with clinical risk stratification identifies a more favourable subgroup than PSMA status alone. At five years, 57% of low-risk patients experienced biochemical progression, indicating that immediate SRT could potentially be deferred in nearly half of these patients.

Several studies report good bPFS rates in patients with negative PSMA PET/CT findings treated with SRT [17,24,26,27]. In addition, Emmett et al. showed that patients with negative PSMA PET/CT findings at BCR had superior biochemical progression-free survival (bPFS) rates after SRT compared to patients with disease on PSMA PET/CT scans. However, our findings indicate that PSAdt and pISUP grade group remain key predictors of progression, even in patients with negative PSMA PET/CT scans. Further stratification is therefore required to identify those likely to benefit from SRT. It is likely that the EAU BCR high-risk patients with a negative PSMA PET/CT scan at BCR, who were likely included in the aforementioned studies, could derive benefit from SRT. Conversely, patients with indolent tumours in those studies might have achieved good outcomes without SRT as well, highlighting the risk of overtreatment. Avoiding overtreatment is particularly important as SRT is associated with genitourinary and gastrointestinal side effects, which can impair quality of life and are only justifiable when clear oncological benefit is expected.

Harsini et al. compared outcomes in 101 patients with negative PSMA PET/CT findings at BCR who were managed with either observation (*n* = 65) or SRT (*n* = 36, with half receiving additional ADT) [25]. Radiological progression occurred in 29% of the observation group and 6% of the SRT group. At three years, bPFS was 71% in the surveillance group versus 79% in the SRT group. While SRT showed better outcomes, this is not surprising as half the patients in the SRT group received additional ADT and/or whole-pelvis radiotherapy. Furthermore, time to progression was not adjusted for ADT use, and no stratification by EAU BCR-risk group was performed.

Celli et al. conducted a prospective study of 103 BCR patients with negative PSMA PET/CT findings and reported 55% radiological progression at a median of 22 months [24]. Significant differences were observed between pathological ISUP grade groups: median time to progression was 20.5 months for pISUP groups 1–2 and 12.1 months for pISUP groups 4–5, with only 9.3% of pISUP group 4–5 patients remaining radiological progression-free at two years compared to 47.8% of pISUP group 1–2 patients. Our findings are consistent with this, with 48% radiological progression at a median of 25 months. However, it is worth noting that some patients in the study by Celli et al., received adjuvant SRT, which may have influenced progression outcomes.

Importantly, Celli et al. reported that 54% of patients with radiological progression had metastatic disease. In our cohort, 74% of PSMA-positive follow-up scans showed metastases. These findings, concordant with prior studies, confirm that most recurrences after a RP are located outside the prostatic fossa, even at low PSA levels [28,29]. This highlights the potential for overtreatment with SRT in patients with negative PSMA PET/CT findings, given the absence of detectable targets. Moreover, in truly low-risk BCR patients, the utility of performing a PSMA PET/CT scan at the time of recurrence may be questionable, as the likelihood of detecting actionable disease appears low.

The benefit of SRT in unfavourable-risk BCR patients is well established. Trock et al. reported a threefold reduction in PCa-specific mortality with SRT (HR 0.32), particularly in men with a PSAdt <6 months [6]. Broeck et al. similarly advocated for early intervention in patients with rapid PSA kinetics [8]. In contrast, our data show that while the EAU BCR-risk classification is predictive for biochemical progression, it does not predict radiological progression or distant metastases. This may reflect the small number of clinical events, as only four distant metastases occurred in the low-risk group, or it may indicate that PSA kinetics alone do not fully capture metastatic potential.

Several limitations must be acknowledged. The observational design and small sample size—particularly in the low-risk subgroup—may limit generalisability and reduce statistical power and the lack of significant differences between risk groups may therefore reflect limited power rather than a true absence of effect. Second, treatment and imaging decisions were not standardised, introducing selection and lead-time bias. Patients were restaged at varying PSA thresholds, with different PSMA tracers (e.g., [^68^Ga]Ga-PSMA-11 and [^18^F]-DCFPyL), and not all patients underwent baseline PSMA PET/CT scans, complicating longitudinal comparisons. Third, the cohort was heterogeneous, including node-positive cases and patients with biochemical persistence aftera RP, potentially skewing outcomes in high-risk groups. Fourth, it is important to note that patients included in this study represent a selected subset, as observational management was not standard practice during the study period. Fifth, follow-up PSMA PET/CT imaging was not performed in all patients, limiting assessment of radiological progression of the full cohort. Finally, evolving EAU guidelines during the enrolment period likely influenced patient selection and follow-up strategies.

## 5. Conclusions

This study assessed oncologic outcomes in patients experiencing biochemical recurrence (BCR) after a radical prostatectomy (RP) who had negative PSMA PET/CT findings and were managed with observation. A prostate-specific antigen doubling time (PSAdt) ≤20 months and a pathological ISUP grade group ≥4 were identified as independent predictors of both biochemical and radiological progression. Patients meeting the European Association of Urology (EAU) criteria for low-risk BCR (a PSAdt > 12 months and a pISUP grade group < 8) had a substantially lower three-year biochemical progression rates (52%) compared to high-risk patients (83%). Radiological progression occurred less frequent in the low-risk group (33%) compared to the high-risk group (55%). These findings suggest that selected patients with negative PSMA PET/CT findings in a BCR setting with favourable clinical features may be safely managed with observation, potentially by deferring salvage radiotherapy (SRT). Integration of PSMA PET/CT imaging with established risk stratification tools may refine patient selection, reduce overtreatment, and support personalised management of BCR. Prospective studies with standardised imaging and treatment protocols are warranted to confirm these findings.

## Figures and Tables

**Table 1:** Baseline characteristics of 89 patients undergoing observational management for biochemical recurrence with negative prostate-specific membrane antigen positron emission tomography/computed tomography findings after a radical prostatectomy.

Variable	All Patients (*n* = 89)
Age at RP (years)	66 (60–69) [46–77]
Initial PSA level (ng/mL)	9.4 (7.3–14) [2.6–182]
Clinical T-stage	
T1c	41 (46)
T2	35 (39)
T3	13 (15)
Biopsy ISUP Grade Group	
1	27 (30)
2	27 (30)
3	14 (16)
4	17 (19)
5	4 (5.0)
EAU BCR-risk classification	
Low	15 (17)
Intermediate	34 (38)
High	40 (45)
Pathology T-stage	
2	56 (63)
3	32 (36)
4	1 (1.0)
Pathological ISUP Grade Group	
1	9 (10)
2	31 (35)
3	30 (34)
4	10 (11)
5	9 (10)
Pathology N-status	
Nx	34 (38)
N0	44 (50)
N1	11 (12)
Pathology R-status	
R0	59 (66)
R1	30 (34)
Biochemical persistence after RP	
No	71 (80)
Yes	18 (20)

*n*: number; RP: radical prostatectomy; PSA: prostate-specific antigen; T-stage: tumour stage; ISUP: International Society of Surgical Pathology grade group; EAU: European Association of Urology; N-status: lymph node status; Nx: no lymph node dissection performed; R-status: surgical margin status. Quantitative variables are expressed as medians; numbers in parentheses are interquartile ranges; numbers in square brackets are ranges. Qualitative variables are expressed as proportions; numbers in parentheses are percentages. Statistical analysis was performed using SPSS, version 28.0.1.1 (IBM, Armonk, NY, USA).

**Table 2:** Clinical characteristics at time of biochemical recurrence in 89 patients undergoing observational management with negative prostate-specific membrane antigen positron emission tomography/computed tomography findings after a radical prostatectomy.

Variable	All Patients (*n* = 89)
Age at BCR PSMA PET/CT (years)	70 (64–72) [47–81]
PSA at BCR	0.2 (0.2–0.3) [0.2–0.7]
PSA doubling time at BCR	10 (5.0–18) [1.0–115]
Time to BCR (months)	23 (11–58) [1.0–163]
EAU BCR-risk group	
Low	27 (30)
High	62 (70)
Follow-up time after BCR PMSA PET/CT (months)	63 (43–83) [36–122]

*n*: number; BCR: biochemical recurrence; PSMA PET/CT: prostate-specific membrane antigen positron emission tomography/computed tomography; PSA: prostate-specific antigen. Quantitative variables are expressed as medians; numbers in parentheses are interquartile ranges; numbers in square brackets are ranges. Qualitative variables are expressed as proportions; numbers in parentheses are percentages. Statistical analysis was performed using SPSS, version 28.0.1.1 (IBM).

**Table 3:** Follow-up outcomes after initiation of observational management in patients with biochemical recurrence and negative prostate-specific membrane antigen positron emission tomography/computed tomography findings following a radical prostatectomy.

Variable	All Patients (*n* = 89)	EAU BCR Low-Risk (*n* = 27)	EAU BCR High-Risk (*n* = 62)	*p*-Value
Follow-up time (mo)	63 (43–83)	53 (39–76)	66 (48–83)	0.09
Biochemical progression				
Yes	74 (83)	16 (59)	58 (94)	
No	15 (17)	11 (41)	4 (6)	
Time to biochemical progression (mo)	13 (9.1–23)	21 (13–35)	12 (7.3–20)	0.01
Radiological progression				
Yes	43 (48)	9 (33)	34 (55)	
No	46 (52)	18 (67)	28 (45)	
Time to radiological progression	25 (14–46)	29 (17–44)	23 (8.5–49)	0.49
Restaging PSMA PET/CT				
Negative	25 (37)	7 (43)	18 (35)	
Local recurrence	11 (16)	3 (19)	8 (15)	
Pelvic LNMs	12 (18)	2 (13)	10 (19)	
Distant metastases	20 (29)	4 (25)	16 (31)	
Total	68 (100)	16 (100)	52 (100)	
Time to distant metastases (mo)	22 (8.6–33)	40 (30–73)	18 (8.0–27)	0.01
Treatment change				
Yes	59 (66)	13 (48)	46 (74)	
No	30 (34)	14 (52)	16 (26)	
Time to treatment change (mo)	17 (9.0–33)	31 (19–45)	15 (9.0–30)	0.01
Start lifelong HT				
Yes	18 (20)	3 (11)	15 (24)	
No	71 (80)	24 (89)	47 (76)	
Time to lifelong HT (mo)	34 (24–55)	47 (30–47)	33 (16–53)	0.42
CRPC				
Yes	6 (7.0)	0 (0.0)	6 (10)	
No	83 (93)	27 (100)	56 (90)	
Time to CRPC (mo)	49 (33–67)	NA	49 (33–67)	NA

*n*: number; EAU: European Association of Urology; BCR: biochemical recurrence; mo: months; PSMA PET/CT: prostate-specific membrane antigen positron emission tomography/computed tomography; LNMs: lymph node metestases; HT: hormonal therapy; CRPC: castration resistant prostate cancer; NA: not applicable. Quantitative variables are expressed as medians; numbers in parentheses are interquartile ranges. Qualitative variables are expressed as proportions; numbers in parentheses are percentages. Statistical analysis was performed using SPSS, version 28.0.1.1 (IBM) and *p* < 0.05 indicated statistical significance. The Mann–Whitney U test was used to compare the medians of two independent groups.

**Table 4:** Biochemical progression rates per year of follow-up, defined as a PSA rise >0.2 ng/mL above recurrence level, stratified by European Association of Urology biochemical recurrence-risk group.

Variable	All Patients (*n* = 89)	EAU BCR Low-Risk (*n* = 27)	EAU BCR High-Risk (*n* = 62)
Progression rate year 1	34/89 (38)	3/27 (11)	31/62 (50)
Progression rate year 2	58/89 (65)	10/27 (37)	48/62 (77)
Progression rate year 3	65/89 (73)	14/27 (52)	51/62 (83)
Progression rate year 4	61/75 (81)	14/24 (58)	47/51 (92)
Progression rate year 5	42/50 (84)	8/14 (57)	34/36 (94)

*n*: number; BCR: biochemical recurrence. Qualitative variables are expressed as proportions; numbers in parentheses are percentages. Statistical analysis was performed using SPSS, version 28.0.1.1 (IBM).

**Table 5:** Cox regression analysis of risk factors for biochemical progression following observational management for biochemical recurrence with negative prostate-specific membrane antigen positron emission tomography/computed tomography findings.

	Univariable		Multivariable	
Variable	HR (95%-CI)	*p*-Value	HR (95%-CI)	*p*-Value
Age (years)			0.98 (0.95–1.02)	0.32
pT3			1.02 (0.62–1.68)	0.94
pISUP ≥ 4			2.04 (1.11–3.74)	0.022
pN-status				0.32
pN0			ref	ref
pN1			1.87 (0.82–4.24)	0.14
pNx			1.60 (0.92–2.77)	0.096
R1-status			1.65 (0.93–2.93)	0.085
Biochemical persistence			1.48 (0.81–2.72)	0.21
PSAdt * (months)	0.14 (0.07–0.29)	<0.01		
PSAdt ≤ 12 months	3.02 (1.80–5.09)	<0.01		
PSAdt ≤ 20 months	6.54 (2.79–15.33)	<0.01	5.72 (2.41–13.56)	<0.01
EAU BCR high-risk	3.10 (1.77–5.44)	<0.01		

HR: hazard ratio; 95%-CI: 95% confidence interval; TIAU: European Association of Urology; pT3: pathological tumour stage 3; pISUP: pathological ISUP grade group; pN-status: pathological lymph node status; R1: positive surgical margins; PSAdt: prostate-specific antigen doubling time; BCR: biochemical recurrence. Statistical analysis was performed using R studio, version 4.3.2, *p* < 0.05 indicated statistical significance. * Continuous values where Log-transformed as variables had moderate to strong Skew (>±0.5).

**Table 6:** Cox regression analysis of risk factors for radiological progression following observational management for biochemical recurrence with negative prostate-specific membrane antigen positron emission tomography/computed tomography findings.

	Univariable		Multivariable	
Variable	HR (95%-CI)	*p*-Value	HR (95%-CI)	*p*-Value
Age (years)			1.01 (0.96–1.06)	0.73
pT3			2.61 (1.23–5.51)	0.012
pISUP ≥ 4			4.45 (2.06–9.65)	<0.01
pN-status				0.084
pN0			ref	ref
pN1			3.24 (1.16–9.11)	0.025
pNx			2.26 (1.11–4.61)	0.024
R1-status			2.39 (1.04–5.50)	0.039
Biochemical persistence			2.80 (1.31–6.01)	<0.01
PSAdt * (months)	0.23 (0.08–0.64)	<0.01		
PSAdt ≤ 12 months	1.31 (0.69–2.48)	0.42		
PSAdt ≤ 20 months	3.57 (1.10–11.55)	0.034	3.44 (1.01–11.74)	0.048
EAU BCR high-risk	1.82 (0.87–3.80)	0.11		

HR: hazard ratio; 95%-CI: 95% confidence interval; iPSA: initial Prostate Specific Antigen; cT: clinical tumour stage; bISUP: biopsy International Society of Urological Pathology grade group; EAU: European Association of Urology; pT3: pathological tumour stage 3; pISUP: pathological ISUP grade group; pN-status: pathological lymph node status; R1: positive surgical margins; PSAdt: prostate-specific antigen doubling time; BCR: biochemical recurrence. Statistical analysis was performed using R studio, version 4.3.2 (Posit Software, Boston, MA, USA), *p* < 0.05 indicated statistical significance. * Continuous values where Log-transformed as variables had moderate to strong Skew (>±0.5).

## Data Availability

The original contributions presented in this study are included in the article. Further inquiries can be directed to the corresponding author.
